# A system for precise analysis of transcription-regulating elements of immunoglobulin genes

**DOI:** 10.1186/1472-6750-5-27

**Published:** 2005-10-04

**Authors:** Emily Y Cheng, Cathy Collins, Maribel Berru, Marc J Shulman

**Affiliations:** 1 Department of Immunology, University of Toronto, Toronto, Canada; 2 Department of Molecular and Medical Genetics, University of Toronto, Toronto, Canada

## Abstract

**Background:**

Precise analysis of expression-regulating elements, such as enhancers and insulators, requires that they be tested under reproducible, isogenic conditions. The commonly used methods of transfecting DNA into cell lines and selecting for drug resistance lack the requisite precision, as they yield cell lines in which varying numbers of gene copies have inserted at varying and undefined sites. By contrast, recombination-mediated cassette exchange (RMCE), by which a site-specific recombinase is used to place a single copy of a transgene at a constant chromosomal site of a cell line, offers the necessary precision. Although RMCE is generally applicable, many regulatory elements of interest are tissue-specific in their function and so require cell lines in the appropriate ontogenetic state.

**Results:**

As reported here, we have used RMCE in a mouse B hybridoma cell line to establish a system with several additional advantages. To avoid the non-physiological features of prokaryotic DNA, this system uses the immunoglobulin μ heavy chain (IgH) gene from the hybridoma as the reporter. Expression can be measured simply by bulk culture assays (ELISA, Northern blot) and single cell assays (flow cytometry). Expression of the IgH reporter gene varies only 1.5 fold among independent transfectants, and expression is greatly (> 50 fold) increased by inclusion of the IgH intronic enhancer.

**Conclusion:**

This system is suitable for precise analysis of the regulatory elements of the immunoglobulin loci.

## Background

Transcription-regulating elements such as enhancers, insulators and silencers are commonly detected by their effects on the expression of transfected genes, i.e., by comparing the expression obtained from transfected DNA that either bears or lacks a candidate DNA segment. Ideally, such comparisons would measure expression in a normal cellular environment and under circumstances in which the only variable is the structure of the transfected gene. However, the commonly used methods do not meet these criteria. Thus, in the case of "transient" transfections, expression is measured one or two days post transfection from extrachromosomal DNA, sometimes at very high copy number. In "stable" transfections, the transfected DNA typically inserts as an array of multiple copies; the insertions occur at undefined and irreproducible chromosomal sites, the copy number varies idiosyncratically, and the multiple copies are in both orientations. These features – insertion site, copy number, and orientation – can affect expression of the transfected DNA and obscure the analysis of regulatory elements. For example, independent transfectants bearing a gene for either the immunoglobulin μ or κ chain showed a 1000 fold range in expression [1,2]. This variation was probably due in part to the effects of neighboring elements at the insertion site [3]. However, the presence of multiple transgene copies in the array is also problematic. On the one hand, the enhancers might act multiplicatively and thus make a weak enhancer appear many fold stronger than reality. On the other hand, repeated copies of the transgene can induce gene silencing, thus leading to an underestimate of enhancer strength [4]. Also, because the array of transfected DNA contains transcription units in tandem and in both orientations, the enhancer lies both 5' and 3' of at least some promoters and in both orientations. This complexity has often obscured whether enhancers in fact function independently of their position and orientation. Finally, many reporter cassettes are derived from bacterial genes, and features such as the relatively high CpG content of non-vertebrate DNA might impose non-physiological requirements on expression.

To analyze regulatory elements in a reproducible (isogenic) context two methods have been used: homologous recombination (HR) and recombination-mediated cassette exchange (RMCE). Expression in such isogenic cell lines typically varies less than two fold [3,5-7]. Although HR has the important advantage that elements are assessed in the normal context, HR carries the disadvantage that the normal locus sometimes contains redundant or counteracting elements that obfuscate analysis. RMCE is useful for analyzing how a specific gene functions at an alternative site. To use RMCE, a selectable/counter-selectable cassette (the target cassette) flanked by site-specific recombination substrates (LoxP or FRT) is placed in the genome, generally at an undefined site [8]. A vector bearing a reporter cassette that is likewise flanked by recombination sites is then co-transfected with a vector expressing the cognate site-specific recombinase (Cre or Flp, respectively). In this way the target cassette is replaced with the reporter cassette, thus always placing the reporter cassette in the same genomic context.

In our earlier work using targeted recombinants to study the role of regulatory elements in the endogenous IgH locus of the mouse, our analysis was impeded by the presence of redundant enhancers [9]. RMCE offered the possibility of overcoming the redundancy problem, thus allowing individual activating elements to be characterized with precision. Because the enhancer and promoter of immunogolobulin genes require B cell-specific transcription factors, the existing RMCE-bearing cell lines could not be used. We therefore established an RMCE system in a mouse (hybridoma) B cell line. As reported here, this system yielded the expected reproducibility and thus allows sensitive, precise measurement of the effects of regulatory elements on gene expression. However, the system did present some unanticipated problems: The predominant product of the counter-selection was not a replacement, and even among the cells with replacements, a significant fraction had undergone additional changes that would obscure or confuse analysis. The additional tests and procedures that we developed to ensure analysis of only those transfectants with the desired replacement are also described here.

## Results and discussion

**Figure 1:**
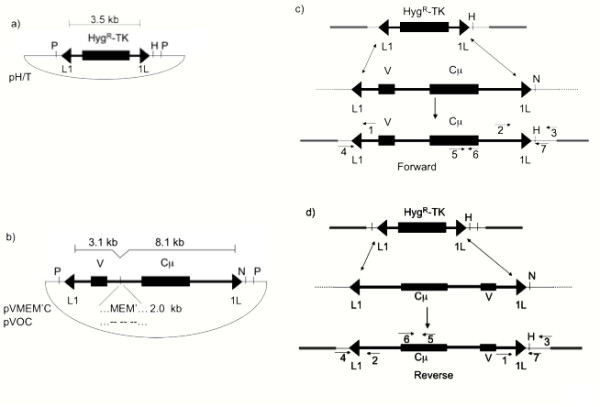
**Structure of the target and reporter cassettes. **In these drawings, the backbone of the vector is represented as a thin line, the cassettes as thick lines, with major exons as rectangles, the LoxP sites as triangles (L1 in the "forward" orientation, 1L in the "reverse" orientation). The three-stranded line represents the chromosomal DNA. The primer sites are indicated by the numbered arrows; numbers refer to the primers listed in Materials and Methods. The figure is not to scale. a) The vector (pH/T) bearing the target cassette. b) The vectors bearing the μ gene replacement cassettes. c) Replacement yielding the μ gene reporter in the forward orientation. d) Replacement yielding the μ gene reporter in the reverse orientation.

The RMCE system described here made use of the Cre recombinase and its cognate LoxP sites and was based on a system developed previously [3]. In this system the selectable/counter-selectable marker was the Hyg^R^-TK fusion gene, which confers resistance to hygromycin (Hyg^R^) and sensitivity to gancyclovir (Gan^S^). In the target vector, which is here denoted as pH/T (Fig. 1), the LoxP sites (denoted L1 and 1L) that flank the Hyg^R^-TK fusion gene are inverted with regard to each other in order to prevent Cre-mediated excision of the target cassette [3].

### Construction of recipient cell line

As noted above, previous work indicated that the enhancer and promoter of immunoglobulin genes require B cell-specific transcription factors. For this reason we established RMCE in a derivative of the mouse B hybridoma cell line, Sp6, which expresses the immunoglobulin μ heavy chain and κ light chain genes at high level and assembles these chains into IgM specific for the hapten, trinitrophenyl (TNP). In designing a system for assessing regulatory elements in the immunoglobulin heavy chain (IgH) locus, we considered that it might be advantageous to use an immunoglobulin heavy chain gene as the reporter. Using the μ gene as a reporter required that the recipient cell line lack a functional μ gene. For this purpose we used a cell line, Z10, that was derived from the Sp6 hybridoma and had deleted the μ gene (see Materials and Methods). μ expression in Z10 could then be measured by ELISA (IgM), flow cytometry (intracellular μ) as well as by Northern blot of μ RNA.

In preparing a derivative of Z10 bearing the target cassette, we sought to minimize the non-physiological DNA that would adjoin the μ reporter and so excised the target cassette with enzymes that cut close to the LoxP sites. Previous work showed that ~40 nucleotides are often excised from the ends of transfected DNA [10], The target vector was therefore cut with Pvu II at sites 152 and 197 nucleotides outside the LoxP sites, with the expectation that cutting in this manner would usually result in transfectants which retained both LoxP sites. As described in Methods, DNA of the target vector was electroporated into the Z10 cell line at a relatively low concentration to reduce the occurrence of tandem or multiple insertions. Transfectants were selected in hygromycin and subcloned. We then confirmed that both LoxP sites were present, using PCR with primer pairs that flanked each of the two LoxP sites, and tested for single copy insertions by Southern blot (data not shown).

### Construction of vectors bearing target and replacement cassettes

To prepare vectors bearing the reporter cassettes, a truncated μ gene from the Sp6 hybridoma was inserted between (inverted) LoxP (Fig. 1). In the vector pVOC the μ gene lacked all the intronic activating elements. The pVMEM'C vector was constructed by inserting the core enhancer (E) and flanking matrix attachment sites (M, M') into pVOC at their normal position. As described below, we used PCR to examine whether the μ gene reporter was intact after replacement. Because the switch region, with its numerous short repeats, could give a variable PCR product and thus interfere with the assessment of the reporter, both replacement vectors were constructed without the switch region.

As illustrated, the target and replacement vectors were identical outside the LoxP sites, except that the HinD III site shown to the right of the LoxP (1L) site in the pH/T vector was changed to a Nhe I site for use in the replacement vectors. This change allowed us to test simply whether the incoming μ gene had become linked to flanking sequences derived from the target vector. Because the two LoxP sites were in opposite orientations, the reporter cassette could recombine in both the "forward" and "reverse" orientations, as illustrated (Fig. 1c, d). The orientations were distinguished by PCR using different primer combinations (see below).

Several potential target cell lines, each bearing a single copy of the target cassette, were then tested for RMCE, i.e., they were co-transfected with the Cre expression vector and a replacement vector. Gan^R ^cells were selected and examined for the replacement cassette. Although none of the candidates yielded replacements with high frequency, one recipient, denoted Z10HyTK2-1, was generally better than the others and was selected for further work.

Another unexpected finding was that of 11 IgM-producing replacements obtained with Z10HyTK2-1, 10 were in the reverse orientation. We do not understand why one orientation was so strongly favored. However, our subsequent work indicated that expression of the μ gene in this orientation was enhancer-dependent and therefore suitable for analyzing enhancer function. The protocol described below was designed to detect replacements only in the reverse orientation and solely on the basis of DNA structure.

### Introduction of the reporter cassette

The recipient cells bearing the target cassette were grown continually in hygromycin to eliminate cells that had spontaneously lost expression of the Hyg^R^-TK cassette. To introduce the replacement cassette, 10^7 ^cells were electroporated in the presence of 50 μg Cre-expression plasmid and 50 μg replacement plasmid. As indicated in Methods, the electroporated cells were divided among multiple flasks to obtain independent replacements. Cells were then incubated in normal medium for 5 days to allow the intracellular pool of Hyg^R^-TK RNA and protein to decrease sufficiently for cells to become resistant to gancyclovir.

Transfectants emerged as large colonies after ~9 days at a frequency of ~1–2 × 10^-5 ^per surviving cell. DNA from these colonies was isolated and subjected to the following four tests to identify proper replacements. The fractions given below for colonies with the indicated features are based on results using several vectors of the same general structure as those shown in Figure 1.

a) Test for a μ gene in the reverse orientation. PCR using primers 1 & 7 detected colonies in which the cells had acquired the μ gene in the reverse orientation (~15% of the total number of Gan^R ^colonies, thus ~1 replacement per 10^6 ^surviving cells).

b) Test for μ gene replacement of the target cassette. The PCR product (primers 1 & 7) was incubated with HinD III and Nhe I to distinguish whether the μ gene had replaced the Hyg^R^-TK gene or inserted elsewhere in the genome. Cutting with only HinD III indicated that the only μ gene in the reverse orientation had replaced the target cassette; cutting with only Nhe I indicated that the only μ gene in the reverse orientation had randomly inserted; cutting with both enzymes indicated that both events had occurred. (4/48 of the μ-containing colonies had random insertions rather than replacements).

c) Test for intact μ gene. For those colonies with a replacement, the DNA was analyzed with two PCRs, using primer pairs 4 & 5 and 6 & 3. These PCRs generated two overlapping DNA segments spanning the entire replacement cassette and flanking LoxP sites. The two PCR products were digested with Xba I and Xba I + HinD III, respectively, and yielded fragments ranging in size from 0.140 kb to 2.9 kb. By comparing these fragments with the digestion products of the original vector we could detect even small alterations. (2/44 of the replacements had undergone a detectable change).

d) Test whether the colony also acquired a randomly inserted μ gene in the forward orientation. For colonies with an intact replacement cassette in the reverse orientation, the DNA was further analyzed by PCR using primer pairs 4 & 6 and 5 & 3. (8/47 of the replacements had an additional, randomly inserted μ gene in the forward orientation).

### Analysis of μ expression

**Figure 2:**
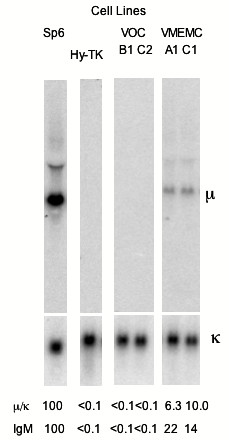
**Analysis of μ expression by Northern blot. **As described in the text, multiple independent replacements were isolated for each vector, and the concentration of IgM in culture supernatant of these cell lines was measured by ELISA. RNA from the cell lines with the highest and lowest IgM concentration was analyzed by Northern blot and probed with segments of the μ and κ genes. The intensity of the bands was quantified by phosphorimager, and the μ/κ ratio, normalized to the value for Sp6 hybridoma, is indicated below each lane. The IgM concentration for each cell line, also normalized to the value for Sp6, is listed below each lane.

**Figure 3:**
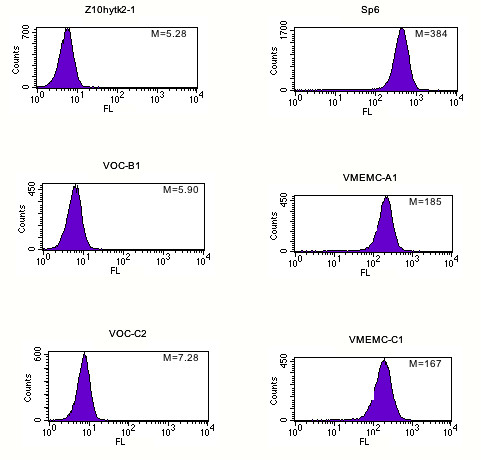
**Analysis of μ expression by flow cytometry. **The indicated cell lines were stained for intracellular IgM using μ-specific, FITC-labeled antibody and analyzed by flow cytometry. Intensity of staining is represented on the horizontal axis, cell number on the vertical. The geometric mean (M) for the staining is noted in each panel.

Using the foregoing protocol we isolated eight independent replacements with vector pVMEM'C and seven with pVOC. Expression of the μ gene was estimated with the IgM-specific ELISA, and the cell lines showing the highest and lowest level of IgM were then analyzed for μ mRNA by Northern blot (Fig. 2) and by flow cytometry of intracellular μ (Fig. 3). In the absence of the enhancer, expression of the μ gene was undetectable with each of the three assays. Each assay indicated that the single copy of the intronic enhancer increased expression of the μ gene by at least 50 fold. As quantified by Northern blot and by ELISA, the reproducibility in expression among independent replacements bearing the μ gene with the intronic enhancer was ~1.5 fold, which is similar to what was reported using this RMCE system and a β-galactosidase reporter in MEL cells [3]. Flow cytometry indicated that expression was homogeneous in the population.

The RNA measurements indicated that the μ gene bearing the intronic enhancer was expressed at ~8% of the level that it was expressed in the original Sp6 hybridoma. According to the measurements by ELISA and flow cytometry (geometric mean) expression of the μ gene in the replacement cassette was ~20% and 40%, respectively, of the endogenous locus. We consider that the Northern blots are the most accurate, as the quantification of the μ and κ RNA by phosphorimager was linear over a large range and normalizing with the μ/κ ratio eliminated variations in input. The difference in μ gene expression between the cassette and the endogenous locus suggests that the endogenous locus includes elements – perhaps the 3' enhancers – that contribute to endogenous expression and were lacking in the replacement cassette.

## Conclusion

The potential advantages of site-specific recombination for constructing isogenic cells were reported many years ago [11,12], and several more refined systems involving the Cre/LoxP system of bacteriophage P1 and Flp/FRT system of yeast have been developed [8]. As reported here, we adapted one such system to a mouse hybridoma B cell line. The system yielded the expected reproducibility (~1.5 fold), and it was therefore a great improvement over other systems in which expression of the μ gene varies 1000 fold. However, using the RMCE system as described here demanded substantially more time and labor than other systems. The extra work was required for two general reasons. First, the frequency of replacement (~15% of the Gan^R ^cells) in our system was lower than what was found previously: > 90% for two lines derived from MEL cells; 10% and 50% for two lines derived from ES cells [3]. Second, our analysis revealed that in a significant fraction (10/47; ~20%) of the cells, the μ gene was not intact or there was an additional, ectopic insertion of the replacement vector. Identifying these cases required specific PCR tests.

Most measurements of the strength of the IgH and other enhancers have been made with transgene arrays, and, as noted in the Background, several features of the array have made it difficult to infer enhancer strength from these measurements. By contrast, our measurement of the effect of a single copy of the IgH intronic enhancer showed that this enhancer increases expression by at least 50 fold. This same system can likewise be used to measure the effectiveness of other regulatory elements – both B cell-specific and tissue-nonspecific – such as promoters and insulators. Inasmuch as this system is amenable to flow cytometry, it will also be useful for studying the molecular basis of variegated expression.

The fact that the reporter is a fully mammalian gene increases the relevance of the measurements. Moreover, because the reporter gene was derived from the endogenous gene of a hybridoma cell line and is expressed in a closely related cell line, the expression of the reporter and the endogenous μ genes can be compared directly to assess the effects of omitting or including individual elements of the endogenous locus in the reporter cassette.

## Methods

### RMCE vectors

The Cre expression vector and LoxP vector were obtained from E. Bouhassira [3]. The structure of the replacement vectors are described in the text and in Figure 1; the nucleotide sequences of the replacement and target vectors are given in the additional files 1–3.

### Solutions

PCR lysis buffer contained 10 mM Tris, pH 8.0, 2.5 mM MgCl_2_, 50 mM KCl, 200 μg/ml gelatin. After autoclaving, this solution was made 0.45% NP40 and 0.45% Tween 20.

### Primers

Numbers 1–7 correspond to the arrows in Figure 1b. Primer 9 was used in place of primer 4 in the initial screening of the target cell lines. Primers 8 and 10 were in the hygromycin-TK target cassette and were paired with primers 7 and 9.

1) (EC35) 5'-GTC TAC GAG GCA AGT GTT-3'

2) (EC68) 5'-AGA CAG TGA CCA GGA GAA GCA A-3'_

3) (CC1L) 5'-GCA CCC CAG GCT TTA CAC TTT ATG-3'

4) (CCL1) 5'-TAA AAC GAC GGC CAG TGA ATT CTC-3'

5) (CCM3) 5'-ATC ATG GAA AGC CAT CCC AAT GGC-3'

6) (CCM4) 5'-TCG TGG CCT ACA ACA CAG GTA TAG-3'

7) (EC69) 5'-AAC AGC TAT GAC CAT GAT TAC G-3'

8) (EC70) 5'-GGA GAT GGG GGA GGC TAA CTG A-3'

9) (EC71) 5'-GAG CCA TAA CTT CGT ATA ATG T-3'

10) (EC73) 5'-GGT CGT TGG GCG GTC AGC CAG G-3'

### Isolation of recipient cell line Z10hytk2-1

The μ gene in the replacement vector was derived from the Sp6 hybridoma cell line, which secretes IgM(κ) specific for the hapten trinitrophenyl (TNP). A mutant of Sp6 lacking the μ gene but expressing the κ light chain was used to assay expression of a transfected μ gene. We planned originally to use a thymidine kinase (TK)-deficient mutant, as in this case bromodeoxyuridine (BrdU) might be used in place of gancyclovir to select replacements. To isolate such a mutant, the TK-deficient cell line igm692-R1, in which the *gpt *gene had been inserted 3' of the endogenous μ gene by gene targeting, was grown in thioxanthine to select Gpt-deficient cells and thus enrich for μ-deficient cells [6,13]. This enrichment was by itself insufficient, so the thioxanthine population was further enriched using the "suicide selection" for IgM deficient mutants [14]. Survivors of this enrichment were cloned and tested for colonies that lacked the μ gene. One such colony, denoted Z10, was used to create the recipient cell line bearing the target cassette. Thus, 1 μg DNA of the target vector was electroporated into 10^7 ^Z10 cells. Hyg^R ^transfectants were selected in 400 μg/ml hygromycin and subcloned. We then tested whether both LoxP sites were present using PCR with primers that flanked the LoxP sites (primer pairs 7 & 8 and 9 & 10 for sites L1 and 1L, respectively). The positive colonies were then tested for single copy insertions by Southern blot. The colonies were not sensitive to BrdU, presumably because this compound is not a good substrate for the fusion protein, and gancyclovir was therefore used. One cell line, denoted Z10hytk2-1, had a comparatively low frequency of spontaneous Gan^R ^cells (1–5 × 10^-5^) and was used in further efforts to improve the protocol and for testing expression of μ replacements.

### Transfection of replacement vector and selection of Gan^R ^colonies

The conditions for electroporation and preparation of cells have been described previously [7]. 10^7 ^recipient cells (Z10HyTK2-1) were washed and resuspended in 0.75 ml PBS and then electroporated with 50 μg Cre expression vector and 50 μg replacement vector, using two pulses at 700 v/0.4 cm and 25 μF. The cells were added to 50 ml normal media and divided among two flasks. Three such electroporations were used for each vector, thus yielding six independent replacements. Survival was measured the following day and was usually ~20%. Incubation in normal medium was continued for 5 days, diluting the cells as needed but ensuring that the cell count remained above 2 × 10^6^/culture. On day 5, cells were plated at 5000 cells/200 μL/well in medium supplemented with 9 μM gancyclovir. On day 14 (9 days after plating) large colonies were evident. These large colonies appeared at a frequency of ~1 × 10^-5^/cell plated for the electroporated cells and ~2 × 10^-6^/cell plated for the control cells. Small colonies appeared in both electroporated and control plates at a frequency of ~2 × 10^-4^/cell plated. Replacements were found only for large colonies.

In an effort to increase the frequency of replacements among the Gan^R ^population of cells, we tried numerous variations in procedure: higher concentrations of hygromycin to select the cells with the target cassette; higher concentrations of gancyclovir to select replacements; different incubation protocols in normal and selective media in the periods before and after transfection. These variations had little, if any, effect.

### Isolation of genomic DNA for PCR analyses

Cells (> 10^4^) were diluted in 1 ml cold PBS and harvested by centrifugation for 1 min at 14000 RPM. The cell pellet was resuspended in 25 μL PCR lysis buffer containing 60 μg/ml proteinase K. This material was incubated for 1 hr at 56°. It was then incubated for 15 min at 95° to inactivate the proteinase K, cooled on ice and used for PCR immediately.

### PCR conditions for testing

#### Presence of both LoxP sites (L1 and 1L) in the recipient cell line

This PCR was done with 1 U Jumpstart Taq (Sigma) in 20 μl Jumpstart buffer, including 0.8 mM dNTP's, 200 ng DNA, and 0.2 μM primers (primer pairs 7 & 8 for 1L; 9 & 10 for L1). Initial denaturation was 94° for 5 min, followed by 36 cycles of denaturation at 94° for 30 sec, annealing at 66° for 30 sec, extension at 72° for 30 sec, and a final extension at 72° for 10 min.

#### Presence of μ gene in forward orientation (Primers 2 & 7)

This PCR was done with 1 U Tsg^+ ^polymerase in 20 μl Tsg buffer including 1.0 mM dNTP's, 100 ng DNA, and 0.2 μM primers (primer pair 2 & 7). Enzyme was added when samples reached 94° for "hot starts." Initial denaturation was at 94° for 3 min, followed by 30 cycles of denaturation at 94° for 30 sec, annealing at 68.5° for 30 sec, extension at 72° for 30 sec, and a final extension at 72° for 10 min.

#### Presence of μ gene in reverse orientation (Primers 1 & 7)

The 20 μl reaction mix in the Pfx amplification buffer contained 1U Platinum Pfx, 3X Platinum Pfx enhancer, 2 mM MgSO4, 2.4 mM dNTP's, 0.6 μM primers. The amplification protocol was an initial denaturation at 94° for 2 min, followed by 32 cycles with denaturation at 94° for 45 sec, annealing at 54° for 30 sec, extension at 68 for 30 sec.

*Integrity of replacement in reverse orientation *(Primers 4 & 5 and 6 & 7). Each reaction tube (25 μl) contained 2 U Roche Expand Long Template enzyme mix, buffer #3, 0.3 μM primers, 2.0 mM dNTP's, 300 ng genomic DNA. Following an initial incubation at 93° for 2 min, the protocol was 10 cycles of denaturation at 93° for 15 sec and extension at 68° for 10 min; for the next 25 cycles the extension time was increased by 20 sec/cycle, with a final extension for 7 min.

*Presence of extra copy of μ gene *(Primers 4 & 6 and 5 & 7). These PCR's used the same conditions as for primers 1 & 7, above.

### Analysis of μ expression

ELISA's and Northern blots were done by standard methods. Flow cytometry of intracellular IgM has been described [7].

## List of abbreviations

Gan^R^, Gan^S^, gancyclovir resistant, sensitive; HR, homologous recombination; Hyg^R^, hygromycin resistant; IgH, immunoglobulin heavy chain; RMCE, recombination-mediated cassette exchange; TNP, trinitrophenyl.

## Authors' contributions

EC and MB constructed the vectors; EC isolated the mutant cell line; EC and CC isolated and analyzed the transfectants; MS provided advice and supervision.

## Supplementary Material

Additional File 1 The additional (document) files denoted pH/T, pVMEM'C, and pVOC are the nucleotide sequences of the corresponding vectors. Click here for file
